# Digital inclusion, legal governance, and the adoption of smart care services by older adults in China: an evolutionary game analysis

**DOI:** 10.3389/fpubh.2026.1912949

**Published:** 2026-09-09

**Authors:** Yu Hu, Yuqi Xiao, Xuan Gao, Zhengzong Huang

**Affiliations:** 1 Law School, Hubei University, Wuhan, China; 2 School of Law, Guangdong University of Technology, Guangzhou, China; 3 Law School & Intellectual Property School, Jinan University, Guangzhou, China; 4 School of Marxism, Shenzhen Technology University, Shenzhen, China

**Keywords:** age-friendly design, digital inclusion, evolutionary game theory, legal governance, smart care services, technology adoption

## Abstract

**Introduction:**

The availability of digital care services does not ensure their adoption by older adults.

**Methods:**

This study develops a tripartite evolutionary game involving government, platform enterprises, and older adults to examine how legal governance, enterprise investment, and user-side benefits jointly shape the adoption of smart care services by older adults in China. The payoff structure applies penalties and reputational losses only to passive enterprise investment and makes age-friendly design, data protection, and support measures improve the relative payoff of adoption. Official procurement records, enterprise disclosures, administrative performance data, subsidy standards, and survey estimates provide direct observable anchors, proxy-informed ranges, or scenario bounds according to parameter identifiability.

**Results:**

Under a coordinated baseline and an initial utilization share of 0.116, the system reached (0.908, 0.919, 0.965) at *t* = 80. A low government-and-enterprise initial state recovered much more slowly but approached the same high-inclusion equilibrium by *t* = 300. Across 2,000 seeded draws, the share with all three strategies above 0.9 increased from 1.95% at *t* = 80 to 6.1% at *t* = 1,000; the high-inclusion corner was locally stable in 6.2% of the drawn parameter sets.

**Discussion:**

These are conditional simulation results, not population probabilities. High inclusion requires the public value of intervention to exceed implementation cost, sanctions and reputation to offset the short-run advantage of underinvestment, and user benefits and support to overcome the outside option.

## Introduction

China's population aged 60 years and above exceeded 310 million at the end of 2024, representing 22.0% of the population (1). Population aging is unfolding alongside rapid digital transformation, making smart care for older adults an increasingly important component of both the silver economy and public-service reform. The national 15th 5-Year Plan (2026–2030) calls for deeper application of digital and intelligent technologies in health, care for older adults, and accessible public services (2). Smart care for older adults is therefore not merely a market for connected devices; it is also a governance setting in which service accessibility, data protection, affordability, and equal participation must be coordinated.

Smart care for older adults uses technologies such as the Internet of Things, big data, artificial intelligence, and mobile platforms to connect service providers with older adults and caregivers. Policy support and market entry have expanded the supply of health monitoring, emergency assistance, telemedicine, and home-based care services (3–5). Yet supply has not translated automatically into use. Older adults remain overrepresented among non-internet users, and adoption is constrained by digital literacy, device and service costs, usability barriers, limited human support, and distrust of data collection and automated services (6, 7).

These barriers have a legal dimension. Health status, biometrics, location, and service-use records may constitute sensitive personal information, triggering duties concerning lawful and necessary processing, transparency, security, and individual rights under China's Personal Information Protection Law (8). The Barrier-Free Environment Construction Law requires the development of accessible information exchange and social services and explicitly connects accessibility with age-friendly adaptation (9). The Law on the Protection of the Rights and Interests of Older Persons further frames participation in social development and access to services as protected interests (10). Accordingly, legal governance in smart care for older adults includes more than punishment: it also encompasses clear accessibility and service-quality standards, data-protection duties, complaint and remedy channels, and positive support for digital inclusion.

The resulting policy problem is strategic. Government decides how intensively to regulate and support the sector; platform enterprises decide whether to invest in age-friendly design, service quality, and data protection; and older adults decide whether the expected benefits justify the financial, learning, and privacy-related costs of adoption. Each decision changes the incentives faced by the other actors. A static account of adoption determinants cannot, by itself, show when these feedbacks produce a low-adoption trap or a high-adoption equilibrium.

This study therefore constructs a tripartite evolutionary game under bounded rationality. Its contribution is not the use of a three-actor game structure alone, which already exists in the literature. Instead, the study integrates government regulation and support, enterprise responsibility for accessibility and data protection, and older adults' economic and psychological adoption benefits in one internally consistent payoff system. It derives analytical stability and threshold conditions, then evaluates them through evidence-informed simulations, service-specific low-utilization initial states, one-parameter threshold tests, and joint range-based sensitivity analysis. The empirical sources bound plausible inputs and observable moments; they do not turn the theoretical model into a causal policy evaluation.

### Literature review

Research on the adoption of smart care services by older adults has developed along three closely related but only partially integrated lines: individual technology acceptance, multi-stakeholder ecosystem coordination, and strategic interaction among public and private actors. Together, these strands show that adoption is not determined solely by older adults' willingness to use technology. It is also shaped by the design and conduct of platform enterprises, the availability of social and community support, and the regulatory and incentive structures established by government. A useful account of the adoption of smart care services by older adults must therefore connect individual-level determinants with the institutional and market processes that produce accessible, trustworthy, and affordable services.

The first strand explains adoption primarily through individual perceptions and capabilities. Technology Acceptance Model (TAM) and Unified Theory of Acceptance and Use of Technology (UTAUT) studies have consistently examined perceived usefulness, perceived ease of use, social influence, facilitating conditions, trust, and technology anxiety. A systematic review found that older adults' technology use is associated with technological, psychological, social, personal, cost-related, behavioral, and environmental antecedents, while also noting the predominance of quantitative and cross-sectional designs (11). A subsequent meta-analysis of 41 studies reported positive associations between behavioral intention and perceived usefulness, perceived ease of use, and social influence, but also found substantial heterogeneity across regions and technology types (12). Research involving Chinese older adults further shows that self-efficacy, anxiety, facilitating conditions, trust, and age-related characteristics can alter adoption pathways (13–15). More recent evidence links digital literacy to smart care adoption through task-technology fit and attitudes toward aging (16). These findings establish that adoption is heterogeneous and context dependent rather than a uniform response to the availability of digital services. Earlier studies of perceived value, satisfaction, cost, caregiver influence, and digital competence support the same multidimensional interpretation (17–22).

Individual acceptance models nevertheless provide only a partial explanation of the adoption of smart care services by older adults. Survey-based TAM and UTAUT models are well suited to estimating associations among user perceptions, intentions, and reported behavior, but they commonly treat regulation, enterprise investment, and service design as fixed external conditions. Configurational methods such as fuzzy-set qualitative comparative analysis show that different combinations of self-efficacy, anxiety, cognitive ability, and health conditions may produce similar adoption outcomes (23), while mixed UTAUT2 and configurational analyses demonstrate the possibility of multiple sufficient pathways to mobile health adoption (24). However, these approaches do not directly trace how government, enterprises, and users revise their strategies in response to one another over time. This limitation is important in smart care for older adults, where changes in regulation or enterprise investment can alter service quality, trust, affordability, and the incentives for subsequent adoption.

The second strand conceptualizes smart care for older adults as a multi-stakeholder socio-technical ecosystem. Research based on symbiosis theory indicates that the evolution of smart care service ecosystems depends on interdependence among resource providers and on the fairness of value co-creation and distribution (25). Value-sensitive design research similarly shows that autonomy, privacy, and personalization may conflict in gerontechnology development, and that incorporating the perspectives of older adults and other stakeholders can improve acceptance (26). Multi-stakeholder studies of dementia care technologies identify interacting technological, financial, ethical, and information barriers, including the often underrepresented role of business actors (27). Research on care robots further demonstrates that adoption is embedded in relationships among older adults, formal caregivers, and family caregivers (28). This literature moves beyond an isolated user perspective and highlights coordination, value alignment, and social support. Yet much of it remains qualitative, case based, or focused on ecosystem description rather than on the strategic thresholds that determine whether collaboration is sustained. A broader review of smart aging research likewise identifies the need to connect technological development with coordinated governance and service delivery (29).

The third strand uses game-theoretic approaches to examine strategic behavior in care for older adults provision and regulation. Existing studies have already developed multi-party evolutionary game models, so the literature cannot be characterized as predominantly single actor. For example, a tripartite model of an intelligent care service platform for older adults shows that value co-creation depends on platform revenue, participation costs, reputation effects, and government subsidies (30). Research on service quality supervision finds that dynamic penalty and subsidy mechanisms can produce different institutional trajectories from static policy instruments, while excessive subsidies may generate unintended behavior (31). A four-party model of Internet-based community care for older adults incorporates government, service providers, platforms, and older adults and identifies threshold effects for penalties, subsidies, and user feedback (32). A rural digital transformation model also demonstrates that regulatory costs, infrastructure expenditure, affordability, reputation, and subsidies affect local governments, service centers, and older adults in different ways (33). Collectively, these studies establish the value of evolutionary game theory for analyzing boundedly rational actors, repeated interaction, multiple equilibria, and policy thresholds. Related work on government supervision and Internet Plus supply games similarly shows that regulatory intensity and comparative returns can redirect strategic evolution (34, 35).

Several studies are particularly close to the present research. Shi et al. modeled the interaction among government regulation, platform privacy protection, and older adults' participation in supervision, identifying privacy losses, platform costs, and rewards and penalties as key determinants of equilibrium (36). Shang and Mi constructed a tripartite evolutionary game involving government, platforms, and older adults to examine the adoption of AI health assistants in smart care for older adults; their findings emphasize adoption costs, platform trust, policy support, and incentive intensity (37). Empirical evidence also suggests that smart care for older adults is positively associated with subjective wellbeing among Chinese older adults, with health status and social activity operating as mediating pathways and digital literacy shaping heterogeneity (38). These studies demonstrate that multi-actor interaction, privacy, trust, and user benefits are already established concerns. They also narrow the contribution that can reasonably be claimed by a new tripartite model.

Across the literature reviewed here, however, the relevant mechanisms are usually examined in separate application-specific models. Individual acceptance research identifies user-level determinants but generally does not endogenize government and enterprise strategies. Ecosystem research captures coordination and value conflict but rarely derives equilibrium conditions. Existing evolutionary games focus on particular problems such as platform construction, service quality, privacy protection, AI assistants, or rural service transformation. As a result, the field still lacks an integrated account of how regulatory and supportive government intervention, enterprise investment in age-friendly design and data protection, and older adults' economic and psychological benefits jointly shape digital inclusion across smart care services for older adults. This gap concerns the integration of mechanisms, not the absence of multi-stakeholder or evolutionary game research.

To address this gap, the present study places government, platform enterprises, and older adults within a unified evolutionary game framework. Government chooses the intensity of regulation and support; platform enterprises decide whether to invest actively in age-friendly design, service quality, and data protection; and older adults choose whether to adopt smart care services for older adults in light of expected benefits, affordability, learning barriers, and trust. The contribution therefore does not lie in introducing a tripartite game structure as such. Rather, it lies in using that structure to connect digital inclusion with legal governance, enterprise responsibility, and user adoption, and to identify the conditions under which their strategies may converge toward a high-adoption equilibrium. Evolutionary game analysis complements, rather than replaces, empirical acceptance research: it can clarify internally consistent strategic mechanisms and threshold conditions, but its policy implications remain model based and require empirical validation.

## Methods

### Scope, actors, and strategy sets

The model represents repeated strategic adjustment among three boundedly rational populations. Government chooses high-level intervention (H) with probability x and low-level intervention (L) with probability 1 – x. High-level intervention combines enforceable accessibility, data-protection, and service-quality requirements with monitoring, complaint handling, digital-literacy support, and targeted incentives. Platform enterprises choose active investment (A) with probability y and passive investment (P) with probability 1 – y. Active investment means investing in age-friendly design, privacy and security safeguards, reliable service delivery, and responsive support; passive investment means underinvesting in one or more of these obligations. Older adults choose adoption (U) with probability z and non-adoption (N) with probability 1 – z. The population interpretation means that x, y, and z are proportions using each strategy, rather than literal probabilities assigned to a single actor.

The model deliberately separates behavioral probabilities (x, y, z) from payoff parameters. The original notation p_1_ and p_2_ is removed because it was used simultaneously as an adoption probability and as a multiplier on fines and losses. Penalties G_1_ and G_2_ now apply only when an enterprise chooses passive investment, with G_1_ > G_2_ ≥0. Active investment incurs neither a non-compliance penalty nor a reputational loss in the payoff matrix. This correction makes the behavioral interpretation and the direction of each incentive consistent across assumptions, payoffs, equations, stability analysis, and simulation.

### Legal-governance mechanisms represented in the model

High-level intervention is an institutional bundle rather than a synonym for a larger fine. The enforcement component represents proportionate sanctions for verified failures in age-friendly adaptation, data protection, or service quality. The supportive component represents digital-literacy programs, affordability assistance, human service channels, and conditional subsidies that reduce compliance or adoption barriers. Accessibility standards correspond to duties to provide usable digital interfaces and retain appropriate non-digital or assisted channels; data-protection duties concern lawful, necessary, transparent, and secure processing of health and other sensitive data; complaint mechanisms allow older adults to report service failures and seek correction or redress. The model aggregates these mechanisms into payoff terms and does not claim to reproduce the full legal doctrine of any statute.

### Payoff parameters and behavioral assumptions

Government obtains a sector-related payoff equal to θ_1_ times enterprise return, where θ_1_ > θ_2_ captures the greater public and fiscal value realized under effective high-level intervention. High intervention has an incremental implementation cost K. When enterprise investment is passive, government also bears a credibility and social-trust loss β_i_L^G^, with β_1_ < β_2_ because monitoring, complaint handling, and corrective action mitigate that loss. Enterprise active investment yields net return R. Passive investment yields short-run return S but is reduced by the applicable penalty G_i_ and, when adopters experience deficient services, by reputational loss T. Thus, an active enterprise is not fined; a passive enterprise is fined according to the intervention regime.

For older adults, adoption provides direct benefit M, while non-adoption provides outside-option payoff N. Active enterprise investment adds design and trust benefit D to adoption. Passive investment imposes loss L_1_ on adopters and L_2_ on non-adopters, with L_1_ > L_2_ because adopters bear learning, switching, privacy, and service-failure exposure. High-level intervention supplies benefit C1 to adopters and C_2_ to non-adopters, where C_1_ > C_2_ ≥ 0 because training, affordability support, and enforceable safeguards are most valuable when the service is actually used. This structure guarantees that, all else equal, active investment raises the relative payoff of adoption rather than lowering it. The resulting payoff structure for all eight strategy combinations is summarized in Table 1.

**Table 1:** Corrected payoff matrix (government, enterprise, older adults).

Strategy	Government	Enterprise	Older adults
H, A, U	θ_1_R – K	R	M + D + C_1_
H, A, N	θ_1_R – K	R	N + C_2_
H, P, U	θ_1_S – K – β_1_L^G^	S – G_1_ – T	M – L_1_ + C_1_
H, P, N	θ_1_S – K – β_1_L^G^	S – G_1_	N – L_2_ + C_2_
L, A, U	θ_2_R	R	M + D
L, A, N	θ_2_R	R	N
L, P, U	θ_2_S – β_2_L^G^	S – G_2_ – T	M – L_1_
L, P, N	θ_2_S – β_2_L^G^	S – G_2_	N – L_2_

### Replicator dynamics

Let ΔG, ΔE, and ΔO denote the payoff advantage of high intervention, active investment, and adoption, respectively. Taking expectations over the other populations' strategies yields:

$$\begin{array}{r} \Delta G\;=\;\left(\theta 1\;-\;\theta 2\right)\left[yR\;+\;\left(1\;-\;y\right)S\right]\;-\;K\;+\;\left(1\;-\;y\right)\\ \left(\beta 2\;-\;\beta 1\right)L\;\\ \Delta E\;=\;R\;-\;S\;+\;xG1\;+\;\left(1\;-\;x\right)\\ G2\;+\;zT\\ \Delta O\;=\;M\;-\;N\;+\;yD\;-\;\left(1\;-\;y\right)\left(L1\;-\;L2\right)\;+\\ x\left(C1\;-\;C2\right) \end{array}$$

The corresponding replicator dynamic system is:

$$\begin{array}{l} dx/dt\;=\;x\left(1\;-\;x\right)\Delta G\\ dy/dt\;=\;y\left(1\;-\;y\right)\Delta E\\ dz/dt\;=\;z\left(1\;-\;z\right)\Delta O \end{array}$$

These equations make the direction of each mechanism explicit. Increasing G_1_ raises the relative payoff of active investment when high intervention is present; increasing R directly raises it; and increasing M directly raises the relative payoff of adoption. Active investment also raises adoption incentives through D and by avoiding the excess passive-investment loss L_1_ – L_2_.

### Equilibrium stability and critical values

At a pure-strategy equilibrium (x^*^, y^*^, z^*^), off-diagonal terms of the Jacobian vanish because each contains x^*^(1 – x^*^), y^*^(1 – y^*^), or z^*^(1 – z^*^). The three eigenvalues are therefore (1 – 2x^*^)ΔG, (1 – 2y^*^)ΔE, and (1 – 2z^*^)ΔO evaluated at that equilibrium. A corner is locally asymptotically stable when all three are negative. In particular, the high-inclusion equilibrium (1,1,1) is stable if and only if the following three inequalities hold:

$$\begin{array}{r} \left(\theta 1\;-\;\theta 2\right)R\;-\;K\;>\;0\\ R\;-\;S\;+\;G1\;+\;T\;>\;0\\ M\;-\;N\;+\;D\;+\;C1\;-\;C2\;>\;0 \end{array}$$

For any interior state, the instantaneous switching thresholds for the focal policy and payoff-difference parameters are obtained by setting the corresponding payoff advantage to zero:

$$\begin{array}{r} G1^{*}=\;\left[S\;-\;R\;-\;\left(1\;-\;x\right)G2\;-\;zT\right]\;/\;x,\;for\;x\;>\;0\\ \delta^{*}=\;xG1\;+\;\left(1\;-\;x\right)G2\;+\;zT,\;where\;\delta \;=\;S\;-\;R\\ M^{*}=\;N\;-\;yD\;+\;\left(1\;-\;y\right)\left(L1\;-\;L2\right)\;-\;x\left(C1\;-\;C2\right) \end{array}$$

Because x, y, and z evolve, these are state-dependent thresholds rather than universal constants. The simulations therefore report both analytical thresholds evaluated at the declared initial state and numerical tipping values under a fixed integration horizon and endpoint criterion.

### Evidence sources, parameter ranges, and numerical procedure

The parameters remain dimensionless payoff scores, but their baselines and ranges are no longer assigned through a uniform perturbation. We distinguish three identification levels. A direct observable anchor is used only when the public record supplies a quantity with an explicit denominator or a directly corresponding behavioral moment. Proxy-informed ranges preserve the sign or relative scale of administrative indicators, firm margins, or standardized behavioral effects without treating them as game payoffs. Scenario bounds are author-specified values used where no public counterfactual identifies the latent payoff. Table 2 labels every parameter accordingly and reports the observable anchor or inferential limitation. This hierarchy improves empirical plausibility without misrepresenting transaction values, satisfaction scores, or structural-equation coefficients as directly estimated utilities (1, 39–53).

**Table 2:** Evidence-informed parameters, ranges, identification status, and sources.

Parameter	Meaning	Baseline [range]	Identification status, evidence, and limitation
θ_1_, θ_2_	Government value coefficients under high/low intervention	0.95 [0.90–0.95]; 0.60 [0.60–0.80]	Scenario bounds, proxy informed: satisfaction and performance grades document administrative quality differences, but no high/low-intervention counterfactual identifies government utility (46, 53)
K	Incremental cost of high intervention	0.12 [0.09–0.165]	Direct monetary anchors with proxy normalization: CNY 90,000 and CNY 165,000 platform security/software assessments relative to a CNY 1 million annual operation contract; cross-city comparability is limited (40, 44, 51)
R, S	Net returns under active/passive investment	R = 0.40 [0.09–0.48]; S = 0.44 [S–R: 0.01–0.06]	R is proxy informed by disclosed positive business margins of approximately 9%−48%; margins are not strategy-specific payoffs. Joint draws sample R from this proxy envelope and set S = R + δ. The one-factor sensitivity holds R at 0.40 and varies the explicit scenario δ = S–R from 0.01 to 0.06, avoiding an inconsistent combination of fixed S and the full R range (41, 42)
β_1_, β_2_	Government trust-loss exposure weights	0.04 [0.03–0.05]; 0.15 [0.10–0.20]	Scenario bounds, proxy informed: issue-order shares, complaints, and satisfaction or performance deductions establish the mechanism but do not identify government trust-loss weights (46, 53)
L^G^	Scale of government credibility loss	0.10 [0.05–0.20]	Scenario only: public performance deficits and complaints motivate the sign, but no observable record identifies the utility scale (46, 53)
G_1_, G_2_	Penalty for passive investment under high/low intervention	0.060 [0.05–0.07]; 0.015 [0.005–0.02]	Contract-informed scenario range: an accessible official procurement document applies 1.5%−2% fee deductions per satisfaction point and 10%−20% deductions for verified service violations; the model uses lower routine and higher credible-sanction scenarios within this observed structure (43)
T	Enterprise reputational/remediation loss	0.06 [0.02–0.10]	Scenario bounds, mechanism documented: termination, non-renewal, remediation, and adverse-record exposure exist, but their payoff loss is not separately estimated (43)
M, N	Adoption benefit and non-adoption outside option	0.22 [0.15–0.30]; 0.36 [0.30–0.45]	Scenario bounds, proxy informed: service-specific utilization of 9.0%−14.4% calibrates z0, while adoption studies constrain signs and relative scale; neither source separately identifies M and N (45, 48–50)
D	Additional benefit from active design and protection	0.15 [0.10–0.32]	Proxy informed: standardized positive effects for usefulness, facilitating conditions, compatibility, trust, and support constrain direction and relative scale but are not converted one-for-one into payoff units (48–50)
L_1_, L_2_	Passive-investment losses for adopters/non-adopters	0.20 [0.15–0.25]; 0.08 [0.05–0.11]	L1 proxy informed by standardized cost, risk, and anxiety effects. L2 is a scenario bound reflecting lower exposure among non-users; the two absolute losses are not separately identified (48–50)
C_1_, C_2_	High-intervention support benefits	0.30 [0.30–0.45]; 0.10 [0–0.10]	C1 proxy informed by official support of CNY 3,000–8,000 per bed/person and CNY 200–300 per month, without treating currency amounts as payoff units. C2 is a scenario for assisted or offline channels and is not independently estimated (47, 52)

**Figure 1:**
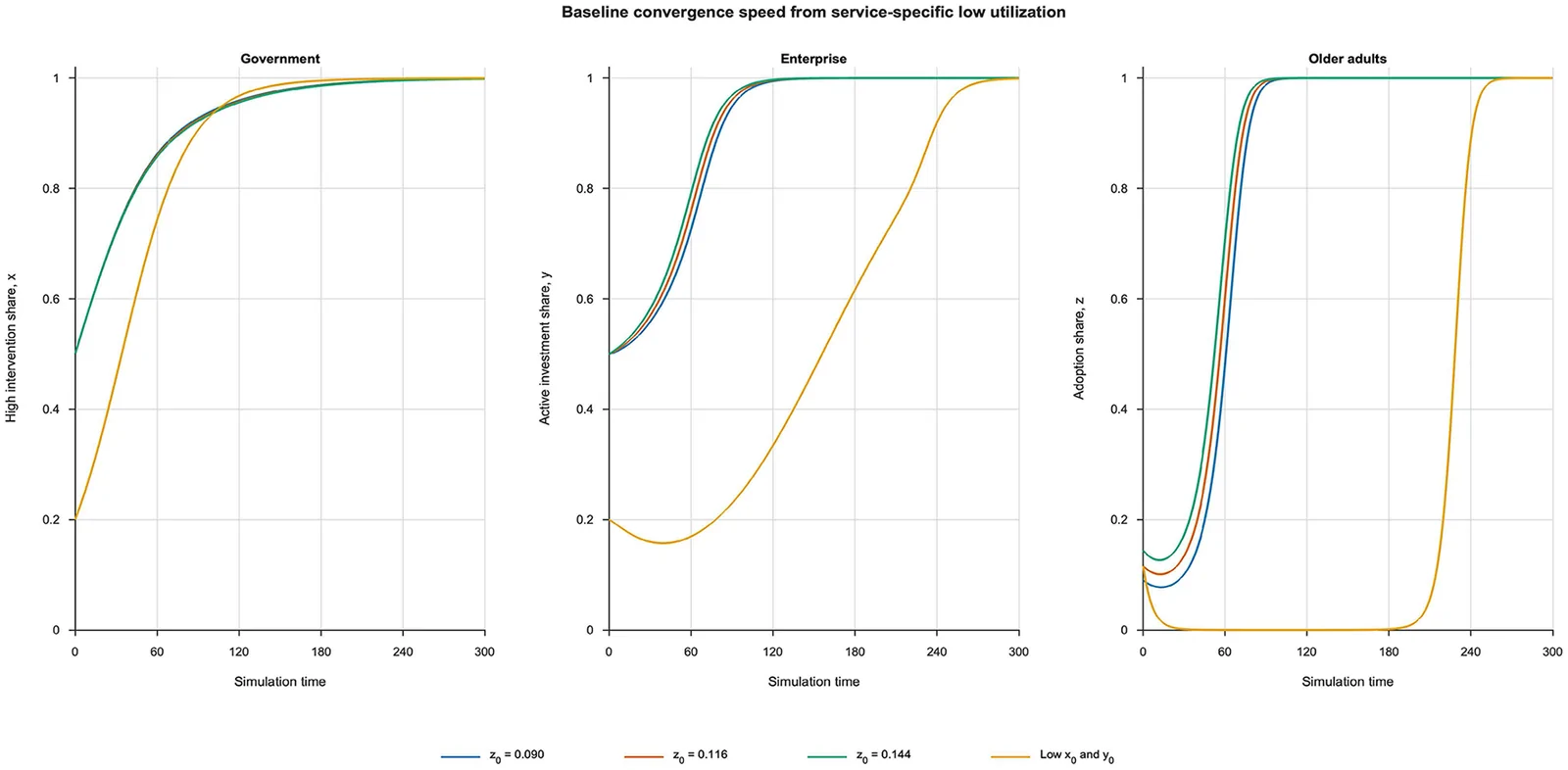
Sensitivity to G1 over the contract-informed scenario range, with zero included as a no-effective-penalty counterfactual. All other parameters remain at the evidence-informed baseline.

**Figure 2:**
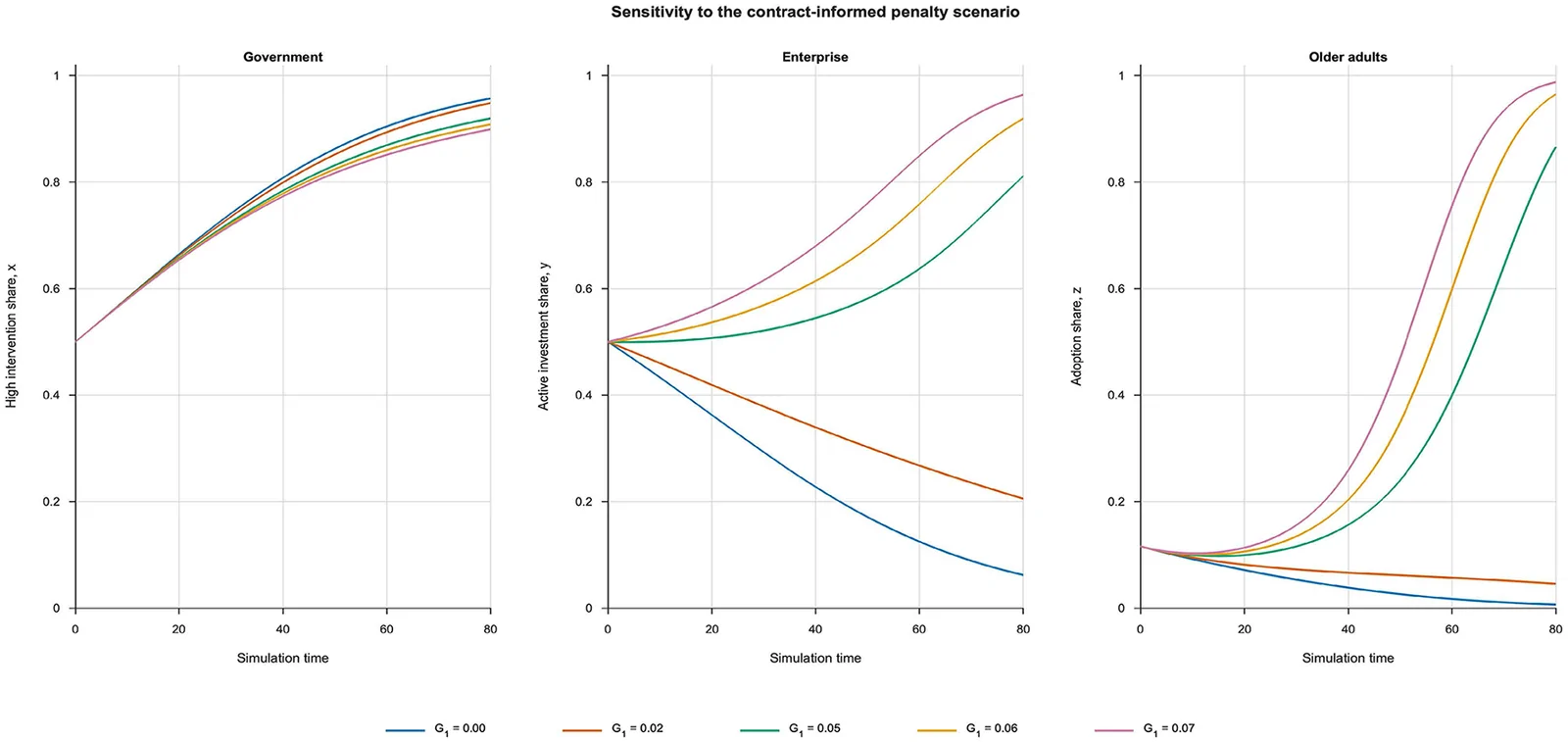
Sensitivity to δ = S – R across the declared 0.01–0.06 short-run passive-investment advantage. R remains 0.40 and S changes with δ; all other parameters remain at the evidence-informed baseline.

**Figure 3:**
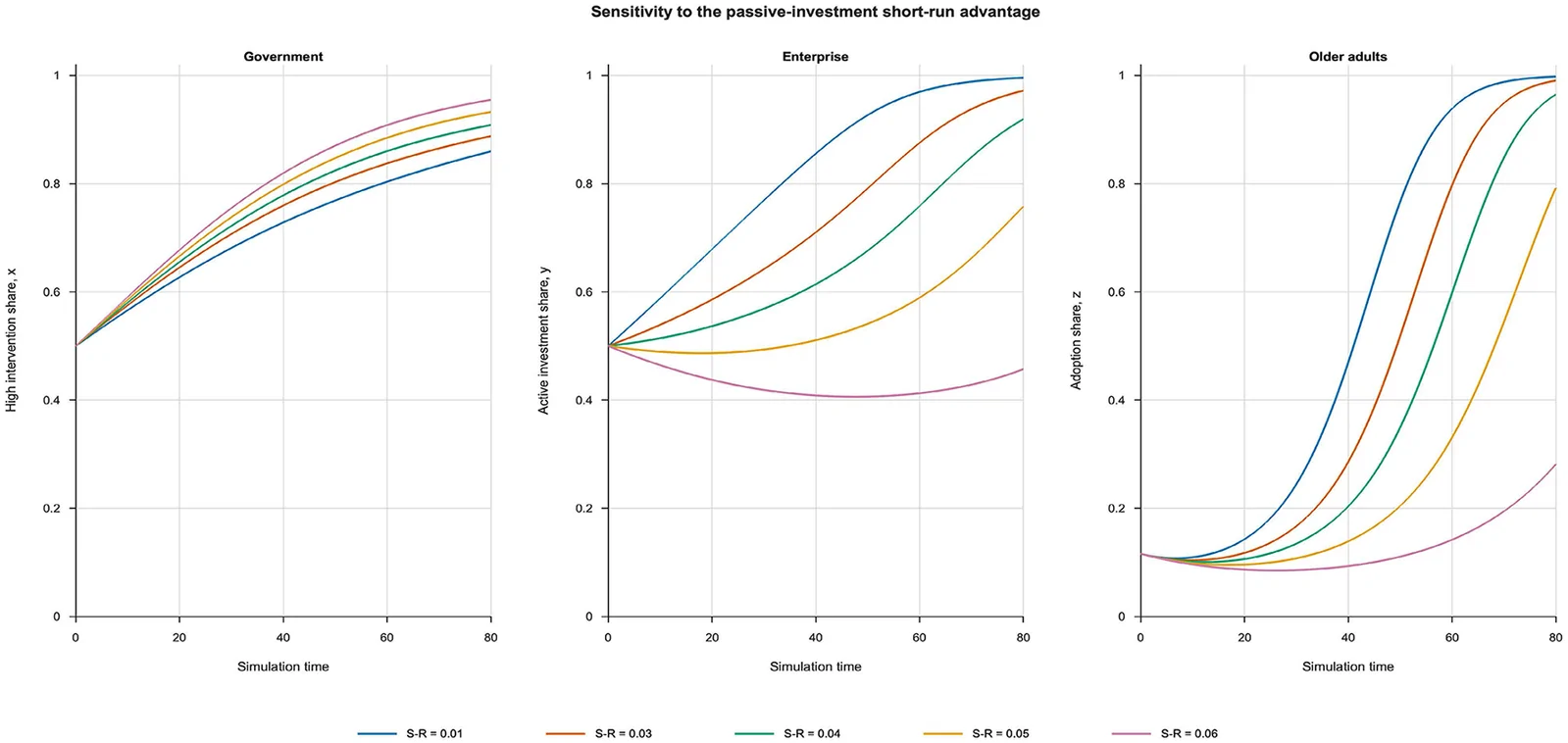
Sensitivity to M across the proxy-informed direct-benefit range. All other parameters remain at the evidence-informed baseline.

**Figure 4:**
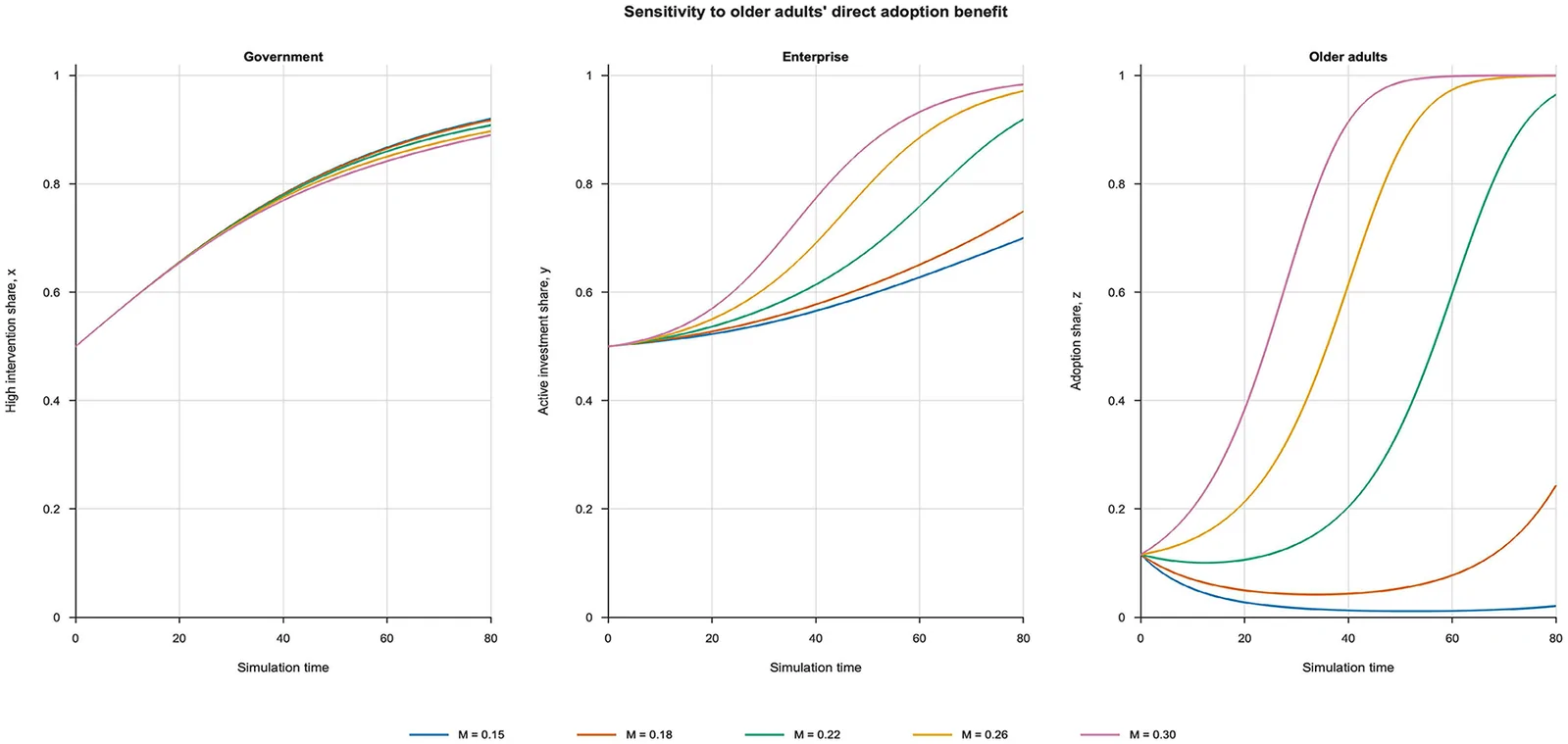
Baseline convergence-speed comparison from service-specific low-utilization starting values and a low government-and-enterprise initial state. Government and enterprise start at x0 = y0 = 0.5 in the first three scenarios; older-adult adoption starts at z0 = 0.090–0.144. The low-participation comparison starts at (0.2, 0.2, 0.116). All trajectories use fourth-order Runge-Kutta integration, step size 0.02, and horizon 300.

The system was solved with a deterministic fourth-order Runge-Kutta algorithm and step size 0.02. Figures 1–3 and the finite-horizon tipping values use *t* = 80. Figure 4 extends the baseline trajectories to *t* = 300 to distinguish slow transient recovery from a different long-run equilibrium. The central baseline uses x0 = y0 = 0.5 and z0 = 0.116; z0 values of 0.090 and 0.144 reproduce service-specific utilization shares reported for three community smart-care services in one cross-sectional sample (45). The asymmetric initialization is intentional: x0 = y0 = 0.5 are neutral interior scenario values because directly comparable observed government and enterprise shares are unavailable, whereas z0 is anchored to reported actual use. Setting z0 = 0.5 would assume 50% initial use, well above the observed service-specific range. Instantaneous thresholds were evaluated at (0.5, 0.5, 0.116), and numerical tipping values were defined by the focal endpoint crossing 0.5 at t = 80. For the enterprise return comparison, the sensitivity parameter is δ = S – R; R remains 0.40 and S changes with δ, preserving the declared short-run underinvestment advantage of 0.01–0.06. The joint sensitivity analysis used 2,000 draws with seed 20, 260, 729. R was drawn from the disclosed positive-margin envelope and S was set 0.01–0.06 higher; x0 and y0 were sampled from U (0.1, 0.9), and z0 from U(0.090, 0.144). Endpoints were evaluated at *t* = 80, 300, and 1,000, and the local-stability conditions at (3, 3, 3) were evaluated directly for each parameter draw. These conditional shares are not empirical occurrence probabilities or reliability coefficients. The solver, fixed seed, archived draws, source data, and a line-by-line MATLAB implementation support computational reproducibility.

## Results

### Analytical equilibrium conditions

The corrected model yields a direct and interpretable condition for the high-inclusion equilibrium. Government maintains high intervention when the additional public value associated with an actively investing sector exceeds the incremental intervention cost. Enterprises maintain active investment when its net return plus the avoided penalty and reputational loss exceeds the passive-investment return. Older adults maintain adoption when direct benefits, active-design benefits, and intervention-related support together exceed the outside option. These conditions explain why no single instrument is sufficient: the government, enterprise, and user inequalities must be satisfied simultaneously.

For compactness, define A^G^0 as the government's payoff advantage from high rather than low intervention at the boundary y = 0, where all enterprises use passive investment: A^G^0 = (θ_1_ – θ_2_)S – K + (β_2_ – β_1_)L^G^. Here, A denotes payoff advantage, the superscript G denotes government, and the subscript 0 denotes y = 0. Table 3 lists the necessary and sufficient local conditions for all eight pure-strategy corners.

**Table 3:** Local stability conditions for the eight pure-strategy equilibria.

Equilibrium	Government condition	Enterprise condition	Older-adult condition
(0,0,0)	A^G^0 < 0	R – S + G_2_ < 0	M – N – (L_1_ – L_2_) < 0
(0,0,1)	A^G^0 < 0	R – S + G_2_ + T < 0	M – N – (L_1_ – L_2_) > 0
(0,1,0)	(θ_1_ – θ_2_)R – K < 0	R – S + G_2_ > 0	M – N + D < 0
(0,1,1)	(θ_1_ – θ_2_)R – K < 0	R – S + G_2_ + T > 0	M – N + D > 0
(0,0,0)	A^G^0 > 0	R – S + G_1_ < 0	M – N – (L_1_ – L_2_) + C_1_ – C_2_ < 0
(1,0,1)	A^G^0 > 0	R – S + G_1_ + T < 0	M – N – (L_1_ – L_2_) + C_1_ – C_2_ > 0
(1,1,0)	(θ_1_ – θ_2_)R – K > 0	R – S + G_1_ > 0	M – N + D + C_1_ – C_2_ < 0
(1,1,1)	(θ_1_ – θ_2_)R – K > 0	R – S + G_1_ + T > 0	M – N + D + C_1_ – C_2_ > 0

For completeness, a fully interior stationary point would require y^*^ = [K – (θ_1_ –θ_2_)S – (β_2_-β_1_)L^G^] / [(θ_1_ –θ_2_)(R–S) – (β_2_-β_1_)L^G^], x^*^ = [N–M+(L_1_-L_2_) – y^*^(D+L_1_-L_2_)] / (C_1_-C_2_), and z^*^ = [S–R–G_2_ – x^*^(G_1_-G_2_)] / T, subject to all three values lying strictly between zero and one. Under the baseline, y^*^ = 1.8, so no fully interior equilibrium is feasible. Moreover, the Jacobian trace is zero at any fully interior stationary point, so it cannot be a hyperbolic locally asymptotically stable sink; a non-hyperbolic case would require higher-order analysis. Additional edge or face stationary points can arise when the relevant remaining payoff advantages vanish; the strict corner conditions in Table 3 remain the relevant tests for the pure high-inclusion outcome.

### Baseline evolution and dependence on initial conditions

Under the evidence-informed coordinated baseline, the three high-inclusion stability margins are positive: (θ_1_ – θ_2_)R – K = 0.020, R – S + G_1_ + T = 0.080, and M – N + D + C_1_ – C_2_ = 0.210. From (0.5, 0.5, 0.116), the endpoint at t = 80 was (0.908, 0.919, 0.965). Initial z0 values of 0.090 and 0.144 produced endpoints (0.911, 0.897, 0.935) and (0.905, 0.936, 0.980), respectively. The low-participation state (0.2, 0.2, 0.116) was only (0.866, 0.204, < 0.001) at *t* = 80, but reached (0.9997, 0.9993, >0.9999) by *t* = 300. Thus, under the baseline parameters, all four interior initial states approach the same high-inclusion equilibrium. Figure 1 shows initial-condition sensitivity in convergence speed and a long low-adoption transient, not a separate low-adoption attractor or basin.

### Analytical and numerical critical values

At the declared initial state (0.5, 0.5, 0.116), the instantaneous thresholds are G_1_^*^ = 0.051, δ^*^ = 0.044, and M^*^ = 0.245, where δ = S – R. The corresponding finite-horizon tipping values were 0.036, 0.059, and 0.191 (Table 4). Differences arise because the other strategy shares evolve before t = 80.

**Table 4:** Thresholds under the evidence-informed baseline.

Parameter	Instantaneous threshold at (0.5, 0.5, 0.116)	Numerical tipping value	Endpoint criterion
G_1_	0.051	0.036	y (*t* = 80) = 0.5
δ = S–R	0.044	0.059	y (*t* = 80) = 0.5
M	0.245	0.191	z (*t* = 80) = 0.5

### Sensitivity to the penalty for passive investment

Within the contract-informed scenario range, increasing G_1_ strengthened active enterprise investment and adoption through the enterprise selection gradient. At G_1_ = 0 and 0.02, y (*t* = 80) was 0.062 and 0.206 and z (*t* = 80) was 0.007 and 0.046. At G1 = 0.05, 0.06, and 0.07, y (*t* = 80) increased to 0.811, 0.919, and 0.964, while z (*t* = 80) reached 0.866, 0.965, and 0.988 (Figure 2). The numerical transition occurred near G_1_ = 0.036 under the declared endpoint criterion. Thus, low and high contract-informed sanction scenarios are not interchangeable: low routine deductions may be insufficient when the short-run return advantage of passive investment persists.

### Sensitivity to the short-run advantage of passive investment

The payoff difference δ = S – R directly measures the short-run return advantage of passive over active investment while preserving the linked parameter constraint used in the joint draws. With R fixed at 0.40 and S = R + δ, increasing δ from 0.01 to 0.06 reduced y (*t* = 80) from 0.996 to 0.457 and z (*t* = 80) from 0.998 to 0.282. At δ = 0.03, 0.04, and 0.05, the endpoints for (y, z) were (0.972, 0.991), (0.919, 0.965), and (0.758, 0.792), respectively. The numerical transition occurred near δ = 0.0586. Government intervention rose as δ increased because passive-sector returns enter the government payoff, but this did not offset the adverse supply-side and adoption effects of a larger underinvestment advantage (Figure 3).

### Sensitivity to older adults' adoption benefits

M remains the direct demand-side bottleneck. At M = 0.15 and 0.18, z (*t* = 80) was 0.021 and 0.244 despite relatively high government intervention. The numerical transition occurred near M = 0.191. At M = 0.22, 0.26, and 0.30, z (*t* = 80) increased to 0.965, 1.000, and 1.000 (Figure 4). Because higher adoption also increases the reputational exposure of passive firms, improving the user's direct value can reinforce enterprise investment rather than act only on demand.

### Joint sensitivity, time-horizon dependence, and local stability

**Figure 5:**
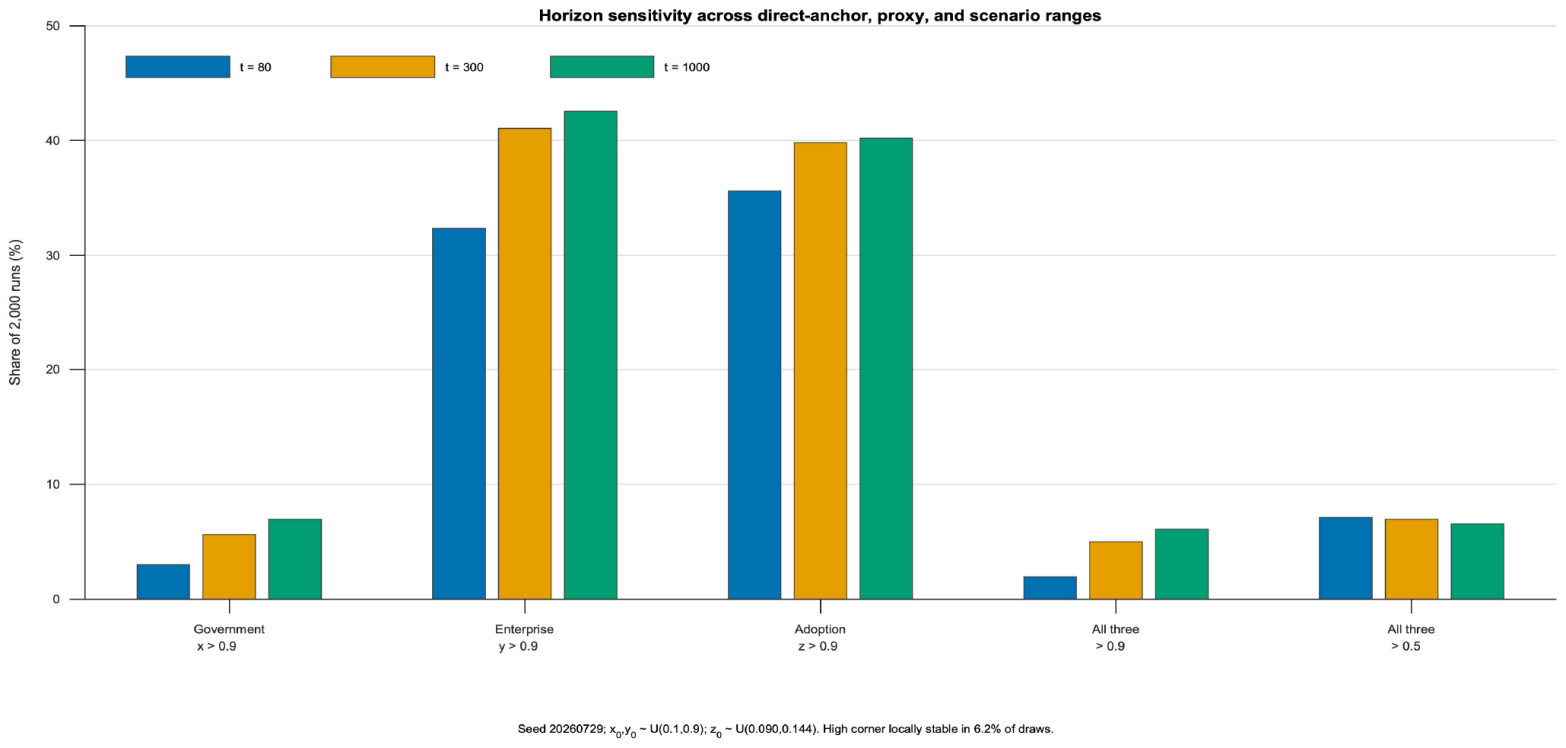
Horizon sensitivity of conditional endpoint shares from 2,000 draws using the prespecified direct-anchor, proxy, and scenario ranges. Initial x and y were sampled from U (0.1, 0.9), and initial z from U (0.090, 0.144). The high-inclusion corner satisfied all three local-stability inequalities in 6.2% of draws.

Across 2,000 seeded draws, the *t* = 80 endpoint shares were unchanged from the prior finite-horizon calculation: x > 0.9 in 3.0%, y > 0.9 in 32.35%, z > 0.9 in 35.6%, all three > 0.9 in 1.95%, and all three > 0.5 in 7.1%. The relevant parameters and their values are summarized in Table 5. Extending the horizon changed the all-three > 0.9 share to 5.0% at *t* = 300 and 6.1% at *t* = 1,000; the corresponding all-three > 0.5 shares were 6.95% and 6.55%. These time-horizon-dependent endpoint shares are illustrated in Figure 5. Direct evaluation of the three analytical stability inequalities showed that (3, 3, 3) was locally stable in 6.2% of parameter draws. The convergence of the *t* = 1,000 high-inclusion share toward this analytical value confirms that the earlier 1.95% figure was a finite-horizon arrival rate, not the size of an attraction basin or a reliability coefficient. The parameter-space restriction is driven mainly by the government condition: the enterprise and older-adult high-corner inequalities were positive in all 2,000 realized draws, whereas the government inequality was positive in only 6.2%.

**Table 5:** Conditional endpoint shares at *t* = 80.

Criterion at *t* = 80	Share of 2,000 runs
x > 0.9	3.0%
y > 0.9	32.35%
z > 0.9	35.6%
x, y, z all > 0.9	1.95%
x, y, z all > 0.5	7.1%

## Discussion

### Interpretation of the corrected mechanisms

The evidence-informed analysis shows a coordination problem with three connected selection gradients. Under the baseline, weak initial government and enterprise participation produces a long low-adoption transient but not a separate attractor: government intervention first rises, enterprise investment responds later, and user adoption recovers last. Under joint parameter variation, different long-run outcomes arise primarily when the public-value gain fails to cover K. This distinction separates slow convergence caused by initial conditions from a genuinely different stable regime caused by payoff inequalities.

This interpretation resolves the counterintuitive results produced by the original payoff matrix. When active enterprises were still charged p_1_G_1_ and γ_1_L_1_, stronger enforcement could mechanically reduce the payoff of the very behavior it was intended to encourage. Once penalties and reputational losses are restricted to passive investment, enforcement increases active investment in the expected direction. Similarly, active design now adds D and removes the excess loss associated with poor services, so an increase in active-investment returns supports rather than suppresses adoption.

### Relation to previous research

The threshold and convergence-speed results are consistent with prior evolutionary-game research showing that subsidies, penalties, platform trust, and participation costs alter strategic trajectories (30–33, 36, 37). The observed 9.0%−14.4% service use and approximately 41%−47% community supply also explain why high stated demand need not produce immediate adoption (45). The present model adds a specific integration: enterprise accessibility and data-protection investment affects adoption through both an added benefit D and avoided user losses, while government intervention combines enforcement with supportive inclusion measures. The range-based results further show that this integration creates a coordination requirement rather than an automatic high-adoption outcome.

Individual technology-acceptance studies identify perceived usefulness, ease of use, trust, anxiety, self-efficacy, facilitating conditions, and social support as important correlates of adoption (11–16, 23, 24). The model does not replace those empirical frameworks. Instead, M, D, L_1_ – L_2_, and C_1_ – C_2_ provide a strategic representation of how some user-level determinants may be altered by enterprise and government choices. The comparison also reveals an important boundary: the model treats each population as internally homogeneous, whereas empirical studies document substantial variation by personal capability, context, technology type, and social support.

### Legal-governance implications

The title's legal-governance dimension is operationalized through five mechanisms. First, data-protection duties require lawful, necessary, transparent, and secure processing of health, biometric, location, and other sensitive information; these safeguards contribute to D and C_1_, while violations support penalties and reputational loss. Second, accessibility and age-friendly standards define what counts as active investment rather than leaving compliance to voluntary corporate claims. Third, service-quality rules and monitoring identify deficient performance. Fourth, accessible complaint, correction, and remedy channels reduce the credibility loss borne by government and make enforcement responsive to actual user experience. Fifth, rights-based digital inclusion requires assisted and non-digital channels so that non-adoption does not become exclusion from essential care. These mechanisms correspond to the policy bundle represented by G_1_, K, βL^G^, C_1_, and C_2_; they should not be collapsed into a single fine.

The empirical ranges sharpen the legal-governance implication. Contract-informed sanction scenarios can redirect enterprise behavior only when they are credible and are not overwhelmed by the short-run savings from underinvestment. Larger statutory fines should be reserved for their specific legal triggers and should not be treated as ordinary service-quality instruments. Conditional procurement and subsidies must also improve the economics of compliant operation, while user-side assistance must raise the realized value of adoption. Complaint data, accessibility testing, privacy incidents, and published performance grades can make these instruments contingent on observable behavior rather than nominal platform entry.

### Practical implications for implementation

The model-based implications support a sequenced policy package rather than a general call for stronger government action:

### Standards and accountable enforcement

Define enforceable minimum requirements for age-friendly interfaces, informed consent, sensitive-data protection, service continuity, and human assistance; apply penalties only after documented non-compliance.

### Conditional supply-side support

Tie innovation subsidies, tax preferences, procurement eligibility, and pilot-program support to audited accessibility, privacy, and service-quality milestones so that public support increases R rather than rewarding passive investment.

### Direct user-side benefits

Increase M and C_1_ through means-tested device or service vouchers, transparent pricing, community digital-literacy programs, trial periods, and trusted in-person support.

### Preserved human and non-digital channels

Retain telephone, counter, caregiver-assisted, and other non-digital channels so that people who do not adopt are not excluded from essential services; this is represented by a viable N and a positive C_2_.

### Accessible complaint and remedy

Establish accessible complaint, correction, data-withdrawal, and redress pathways, and publish aggregated performance indicators to strengthen trust while avoiding disclosure of personal information.

### Heterogeneity-aware evaluation

Evaluate rural–urban, income, disability, health-status, education, caregiver-support, and prior-experience differences before scaling a single intervention package nationwide.

These recommendations are implications of the model and the declared evidence mapping. Official contracts and administrative records support the scale of several inputs, but the proxy-calibrated latent utilities are not causal estimates of policy effects. The range-based analysis is therefore used to identify feasible and fragile regimes, not to claim a nationally optimal fine, subsidy, or adoption target.

### Limitations and future research

Six limitations bound the conclusions. First, older adults are represented as one population even though income, education, health status, disability, rural-urban location, caregiver support, and prior technology experience may change payoffs and learning. Second, government and platform enterprises are aggregated despite large differences in jurisdictional scale, business model, and fixed costs. Third, the ranges combine directly observed monetary or contractual quantities with proxy-calibrated latent utilities; multiple parameter combinations can reproduce the same endpoint, so the payoff vector is not uniquely identified. Fourth, cross-city procurement amounts and enterprise margins are normalized to common scales but do not constitute a matched panel. Fifth, finite-horizon endpoint classifications depend on the selected simulation time, as the *t* = 80, 300, and 1,000 comparison demonstrates. Sixth, the deterministic replicator system assumes continuous adjustment and fixed parameters, excluding shocks, delayed enforcement, networks, and strategic misreporting.

Future research should link platform records, complaint and sanction data, enterprise project accounts, and longitudinal user behavior within the same jurisdiction. Discrete-choice experiments or expert elicitation can estimate latent payoff differences that administrative records cannot identify. Multi-population extensions should distinguish older adults by digital literacy, income, disability, geography, and caregiver support, and enterprises by compliance cost and service model. Agent-based or stochastic models could incorporate network learning, shocks, and delayed enforcement.

## Conclusion

This study provides an internally consistent tripartite evolutionary game of the adoption of smart care services by older adults in which government intervention, enterprise investment, and older adults' adoption respond to distinct but connected payoff advantages. Correcting the payoff matrix eliminates penalties and trust losses for active enterprises and makes age-friendly, privacy-protective investment increase the relative payoff of adoption. The analytical results show that a high-inclusion equilibrium requires three conditions at once: the public value of high intervention must exceed its incremental cost, active investment plus avoided sanctions and reputational loss must outperform passive investment, and the benefits of adoption and support must exceed the outside option.

Evidence-informed simulations show that a coordinated baseline can move from service-specific low utilization toward a high-inclusion state. A low initial government-and-enterprise state delays adoption substantially but approaches the same baseline equilibrium over a longer horizon. Across the declared parameter ranges, only 6.2% of draws satisfy all three local-stability conditions for the high-inclusion corner, and 6.1% reach all three shares above 0.9 by *t* = 1,000. The appropriate conclusion is therefore conditional: enforcement, viable returns to accessible and privacy-protective investment, and direct affordability and support for older adults must jointly satisfy their respective thresholds. The model identifies feasible and slow or unstable regimes; it does not establish a universal optimal policy level or an empirical probability of nationwide adoption.

## Data Availability

The original contributions presented in the study are included in the article/supplementary material, further inquiries can be directed to the corresponding author.
